# Supplementary material for: Causal Relationship between Aging and Anorexia Nervosa: A White-Matter-Microstructure-Mediated Mendelian Randomization Analysis
Source: Biomedicines. 2024 Aug 16;12(8):1874. doi: 10.3390/biomedicines12081874 (PMC11351342; doi:10.3390/biomedicines12081874)
Supplement: Supplementary file 1 [file biomedicines-12-01874-s001.zip › Supplementary Figure/Figure S4/Singe SNP plot of MR analysis for TL to ubm-b-1561.pdf]

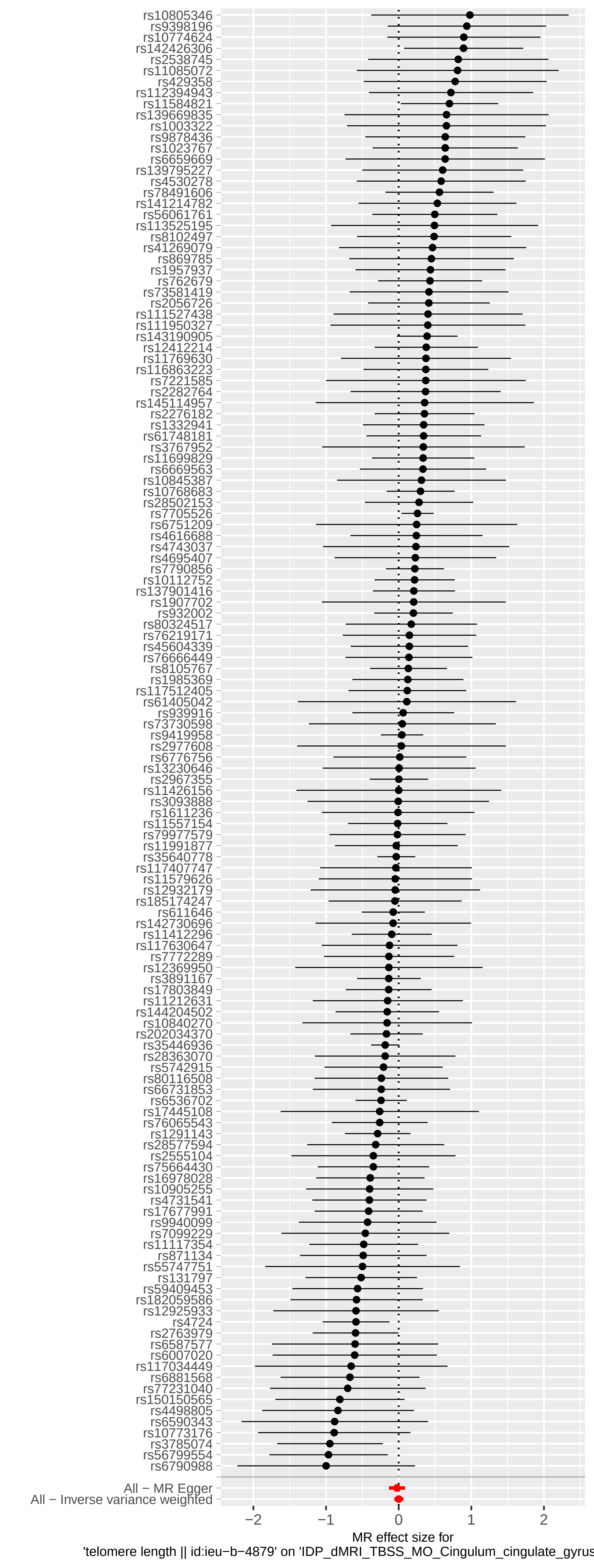
rs10805346
rs9398196
rs10774624
rs142426306
rs2538745
rs11085072
rs429358
rs112394943
rs11584821
rs139669835
rs1003322
rs9878436
rs1023767
rs6659669
rs139795227
rs4530278
rs78491606
rs141214782
rs56061761
rs113525195
rs8102497
rs41269079
rs869785
rs1957937
rs762679
rs73581419
rs2056726
rs111527438
rs111950327
rs143190905
rs12412214
rs11769630
rs116863223
rs7221585
rs2282764
rs145114957
rs2276182
rs1332941
rs61748181
rs3767952
rs11699829
rs6669563
rs10845387
rs10768683
rs28502153
rs7705526
rs6751209
rs4616688
rs4743037
rs4695407
rs7790856
rs10112752
rs137901416
rs1907702
rs932002
rs80324517
rs76219171
rs45604339
rs76666449
rs8105767
rs1985369
rs117512405
rs61405042
rs939916
rs73730598
rs9419958
rs2977608
rs6776756
rs13230646
rs2967355
rs11426156
rs3093888
rs1611236
rs11557154
rs79977579
rs11991877
rs35640778
rs117407747
rs11579626
rs12932179
rs185174247
rs611646
rs142730696
rs11412296
rs117630647
rs7772289
rs12369950
rs3891167
rs17803849
rs11212631
rs144204502
rs10840270
rs202034370
rs35446936
rs28363070
rs5742915
rs80116508
rs66731853
rs6536702
rs17445108
rs76065543
rs1291143
rs28577594
rs2555104
rs75664430
rs16978028
rs10905255
rs4731541
rs17677991
rs9940099
rs7099229
rs11117354
rs871134
rs55747751
rs131797
rs59409453
rs182059586
rs12925933
rs4724
rs2763979
rs6587577
rs6007020
rs117034449
rs6881568
rs77231040
rs150150565
rs4498805
rs6590343
rs10773176
rs3785074
rs56799554
rs6790988
All − MR Egger
All − Inverse variance weighted
−2
−1
0
1
2
MR effect size for
'telomere length || id:ieu−b−4879' on 'IDP_dMRI_TBSS_MO_Cingulum_cingulate_gyrus
